# Integrated sRNA-seq and RNA-seq Analyses Reveal a microRNA Regulation Network Involved in Cold Response in *Pisum sativum* L.

**DOI:** 10.3390/genes13071119

**Published:** 2022-06-22

**Authors:** Mélanie Mazurier, Jan Drouaud, Nasser Bahrman, Andrea Rau, Isabelle Lejeune-Hénaut, Bruno Delbreil, Sylvain Legrand

**Affiliations:** 1 BioEcoAgro Joint Research Unit, Université de Lille, INRAE, Université de Liège, Université de Picardie Jules Verne, 59000 Lille, France; melanie.mazurier@gmail.com (M.M.); nasser.bahrman@gmail.com (N.B.); bruno.delbreil@univ-lille.fr (B.D.); 2 BioEcoAgro Joint Research Unit, INRAE, Université de Lille, Université de Liège, Université de Picardie Jules Verne, 80200 Estrées-Mons, France; jan.drouaud@inrae.fr (J.D.); andrea.rau@inrae.fr (A.R.); isabelle.lejeune-henaut@inrae.fr (I.L.-H.); 3 Université Paris-Saclay, AgroParisTech, INRAE, GABI, 78350 Jouy-en-Josas, France; 4 Univ. Lille, CNRS, UMR 8198—Evo-Eco-Paleo, 59000 Lille, France

**Keywords:** pea, cold stress, cold acclimation, miRNA, RNA-seq, sRNA-seq

## Abstract

(1) Background: Cold stress affects growth and development in plants and is a major environmental factor that decreases productivity. Over the past two decades, the advent of next generation sequencing (NGS) technologies has opened new opportunities to understand the molecular bases of stress resistance by enabling the detection of weakly expressed transcripts and the identification of regulatory RNAs of gene expression, including microRNAs (miRNAs). (2) Methods: In this study, we performed time series sRNA and mRNA sequencing experiments on two pea (*Pisum sativum* L., *Ps*) lines, Champagne frost-tolerant and Térèse frost-sensitive, during a low temperature treatment versus a control condition. (3) Results: An integrative analysis led to the identification of 136 miRNAs and a regulation network composed of 39 miRNA/mRNA target pairs with discordant expression patterns. (4) Conclusions: Our findings indicate that the cold response in pea involves 11 miRNA families as well as their target genes related to antioxidative and multi-stress defense mechanisms and cell wall biosynthesis.

## 1. Introduction

Grain legumes produce seeds that are a valuable source of protein and starch for both animal and human diets [1,2]. Additionally, these crops can establish a symbiosis with nitrogen-fixing soil bacteria, allowing a reduction in the use of nitrogen fertilizers in cropping systems and thus N_2_O emissions [3]. Due to these agronomical and ecological benefits, legumes are considered to be a key component of sustainable agriculture [4,5]. Among legumes, dry peas (*Pisum sativum* L., *Ps*) are widely grown in cool temperate areas. Although they are usually sown in spring in Northern Europe, autumn sowings (winter peas) are desirable as they allow for an increased and stabilized seed yield, as a result of a longer life cycle and the possibility of avoiding frequent drought during the reproductive period [6]. Frost is the main abiotic stress that must be overcome during the winter period. Thus, breeding for winter varieties has motivated several studies aiming at localizing the genetic determinants of frost tolerance and understanding the molecular bases of this trait [7,8,9,10,11]. In these studies, special attention has been given to the ability of frost tolerant genotypes to acclimatize to cold, that is to improve their frost tolerance level in response to the low above-zero temperatures generally encountered in autumn in field conditions. Understanding the mechanisms that regulate the cold acclimation process is of growing importance in the context of climate warming, in which plants must face milder autumns, less favorable to cold acclimation, and alternating acclimation and deacclimation periods.

Recently, next generation sequencing (NGS) technologies have opened new opportunities to understand the molecular bases of stress resistance, such as cold tolerance, notably by allowing the detection of weakly expressed transcripts and the identification of regulatory RNAs of gene expression, such as microRNAs (miRNAs). Among small RNAs (sRNA), miRNAs are a class of molecules of ~19–24 nt that silence the expression of their target genes through the cleavage or inhibition of mRNA translation. In plants, both mechanisms require an almost perfect base pairing between miRNAs and their mRNA targets [12,13]. miRNAs are known to be major regulators of developmental processes and of biotic and abiotic stress responses in plants, as summarized in recent articles [14,15,16]. Among them, plant miRNAs identified in temperature stress response have been the subject of dedicated reviews [17,18]. Concerning the cold response in particular, although some miRNAs appear to be species-specific, twelve of them have been repeatedly identified in various model plant or major crop species, including miR156, miR164, miR166, miR167, miR169, miR171, miR319, miR394, miR395, miR397, miR398 and miR408 [18]. Although some conserved target genes of these miRNAs have also been identified, the cold regulation network involving miRNAs may also vary to some extent between plant species and may moreover interact with miRNA regulation of plant development and response to biotic stresses [15,16]. Despite this relative complexity, some cold responsive miRNAs appear to be potential candidates for cold and frost tolerance improvement, as evidenced by studies of genetically engineered plants (*Arabidopsis thaliana*, *At* [19]; *Nicotiana tabacum*, *Nt* [20]; *Citrus limon* [21]).

In this study, we report the differential expression of miRNAs and their mRNA targets during the cold acclimation of two contrasted pea genotypes, a frost-tolerant line (Champagne; Ch) and a frost-sensitive one (Térèse; Te). This analysis allowed us to provide a comprehensive view of miRNA-mediated regulations during cold stress response in pea. We produced a total of 24 sRNA libraries from either Ch or Te, submitted or not to a period of cold exposure, over three different time points. In addition, we took advantage of the recent release of a pea reference genome (cv Cameor, [22]) to predict a total of 136 miRNAs, for which the expression level was established in each of the experimental conditions. The comprehensive set of annotated genes in the reference genome was used for miRNA target prediction. This enabled us to perform a new assessment of gene expression during cold acclimation, using RNA-seq data previously produced by Bahrman et al. [11]. More particularly, this integrative transcriptomic analysis allowed us to identify 39 miRNA/mRNA target pairs exhibiting discordant expression patterns. A functional investigation of this regulation network highlighted the roles of the miR156, miR159, miR166, miR167, miR396, miR397, miR482, miR2111, miR5770 and miR5232 families and of their putative target genes in cold response in pea. Antioxidative and multi-stress defense mechanisms were particularly highlighted, as well as lignin polymerization through the regulation of the laccase enzyme family.

## 2. Materials and Methods

### 2.1. Experimental Design and Small RNA Sequencing

Twenty-four samples were harvested as described in [11]. Briefly, 2 pea lines, namely Ch, frost tolerant, and Te frost sensitive, were grown under contrasted temperature conditions within 2 experiments. After an identical nursery period (21 days at 20 °C day/14 °C night), plants were exposed either to a low temperature treatment (LT; 16 days at 8 °C day/2 °C night, i.e., a daily average temperature of 4.5 °C), or to an extended nursery period (N; 6 more days at 20 °C day/14 °C night). During the low temperature (LT) or control (N) treatment, plants were sampled 3 times (T0, T1 and T2), with 2 biological replicates. In both experiments, T1 corresponded to 386 degree-days, i.e., T0 + 36 degree-days and T2 corresponded to 472 degree-days i.e., T0 + 122 degree-days (see Additional File S1 for a schematic representation of the study design).

Total RNAs from each sample were sent to Eurofins MWG GmbH where small cDNA libraries with insert size of 19–30 bp were prepared. Subsequently, the 24 bar-coded libraries were sequenced with an Illumina HiSeq 2000/2500 machine, leading to a total of approximately 380 million single-end 50 bp reads. Raw sRNA-seq data were transferred to the NCBI-SRA database, in the BioProject PRJNA543764 [23], which also includes RNA-seq data previously deposited.

### 2.2. sRNA-seq Data Pre-Processing

Adapters were removed from the Illumina reads using Cutadapt (v1.2.1; [24]) and reads were cleaned using Prinseq (v0.20.4; [25]) with the following parameters: -min_len 18 -max length 25 -min_qual_mean 25 -trim_qual_right 20. The quality of the Illumina cleaned reads was checked using FastQC (v0.10.1; [26]). rRNAs, tRNAs, snRNAs and snoRNAs were removed from the sequences through Bowtie alignment (v.1.1.2; [27]) using a set of 7743 eukaryotic sequences obtained from the NCBI database.

### 2.3. Identification of miRNAs and Their Targets

Prior to miRNA detection using miRkwood (v1.0; [28]), sRNA sequences were mapped without mismatch on the Cameor pea genome [22] and on the Ch/Te pea transcriptome [11] using Bowtie (-v 0 --all --best --strata). miRkwood was used with default parameters and only predictions with a score ≥5 (maximum of 6) were kept. The quality score provided by miRkwood (from 0 to 6) arises from the 6 criteria used to detect miRNAs. The first criterion is related to the stability of the stem-loop structure of the precursor, whereas the five others are related to the distribution of mapped reads along the putative pre-miRNA. They allow us to determine whether this distribution presents a typical profile with 2 peaks, corresponding to the guide and the passenger miRNAs. Candidates with a score of 6 fulfill all criteria, whereas those with a quality score of 5 fail a single criterion.

miRNA predictions obtained using separate analyses for Ch and Te samples by aligning reads on either the pea genome or the pea reference transcriptome were then merged. Conserved miRNAs were identified by comparing miRNA predicted sequences to the 10,414 mature plant (Viridiplantae) miRNAs deposited in miRBase (v22; [29]) using Exonerate (v.2.2.0; [30]) selecting only alignments with at most three mismatches. Finally, only miRNA predictions with a miRkwood score of 6 or those with both a score of 5 and showing alignments with miRNA sequences from miRBase were considered for further analyses. miRNA sequences were additionally compared to the 68 miRNAs identified by Kreplak et al. [22] in developing seeds; only exact matches between miRNA sequences were considered.

For every conserved and non-conserved miRNA, targets were predicted using TargetFinder (v1.7; [31]) with default parameters and the pea predicted coding sequences as the target sequence database.

### 2.4. RNA-seq Data Processing

For each RNA-seq library from Bahrman et al. [11], the overall quality of reads was checked using FastQC (v0.11.4; [26]). Adapter sequences were removed using Cutadapt (v1.0; [24]). For every sequenced fragment (i.e., pair of reads), overlapping ends were merged into a single sequence and nucleotides with the lowest quality score were replaced by an N until the overall error rate of the read dropped below 2%. N at read ends were also discarded.

The resulting fragments (either pairs of reads or merged pairs) were aligned to the Cameor genome sequence (v1.0; [22]) using HISAT (v2.2.1.0; [32]), with the following command line: hisat2 -x <genome-index> --n-ceil L,500,0 --np 0 --max-intronlen 20,000 --score-min L,0,-0.5 --known-splicesites-infile <ss-file> -k 100. The resulting files were further processed to remove reads with multiple alignments. These unambiguous alignments were finally used to build a table of gene counts.

All RNA-seq data processing steps were performed with a Python script, using the Python Standard Library and Biopython [33], HTSeq [34], and Pysam [35] (https://github.com/pysam-developers/pysam accessed on 22 April 2020).

### 2.5. Differential miRNA and Gene Expression Analysis

Differential expression analyses were performed with the R (v4.0.3)/Bioconductor (v3.12) package edgeR (v3.32.1; [36]). Non-uniquely mapped reads and genes with less than one count per million in at least half of the twenty-four libraries were filtered out. mRNA and miRNA library sizes were normalized using the TMM approach [37]. Differentially expressed genes (DEGs) and differentially expressed miRNAs were identified by a negative binomial generalized linear model (GLM) using a group-means parameterization (where each combination of genotype, treatment, and time is defined as a group) and the Cox-Reid profile-adjusted likelihood method for estimating dispersions. Comparisons of interest were then estimated using contrasts, using a false discovery rate (FDR) adjusted *p*-value < 0.05 as a significance threshold. T1 and T2 DEGs and differentially expressed miRNAs were identified within each time point by comparing the LT to the N condition for each genotype (Ch or Te) (Additional File S1).

For both periods, we identified four sets of DEGs and differentially expressed miRNAs: (1) between LT and N in Ch at T1; (2) between LT and N in Ch at T2; (3) between LT and N in Te at T1; and (4) between LT and N in Te at T2. These sets were further used to pinpoint Ch- and Te-specific cold responsive genes and miRNAs as well as those involved in cold response common to both genotypes. T0 was treated as a control time point, since environmental conditions for both the LT and N experiments were identical until this sampling date. As such, mRNAs or miRNAs with unexpected differential expression between the LT and N conditions at T0 were removed from the lists of differential elements of interest.

### 2.6. Gene Ontology Enrichment Analysis

The GO enrichment analyses for DEGs and miRNA targets were performed with the R (v4.0.3)/Bioconductor (v3.12) package topGO (v2.42.0; [38]). The elim algorithm with a Fisher test and a nodeSize at 5 was used to perform the analysis. The GO annotation file described in [22] was used to build the mapping between GO terms and Cameor genes. In the visual representation of enrichment analysis results, GO terms were clustered according to their semantic similarity calculated with GOGO [39] using hierarchical clustering with complete linkage.

## 3. Results

### 3.1. Small RNA Analysis

**Figure 1:**
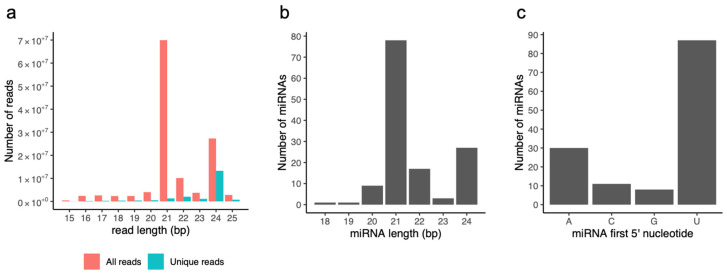
Features of sRNA reads and miRNA predictions. (**a**) Distribution of Illumina read lengths for all (red) and unique (blue) sRNA reads. (**b**) Distribution of miRNA lengths. (**c**) Number of miRNAs initiating with a 5′ A, C, G, or U.

High-throughput Illumina sequencing yielded between 9.9 and 27.6 million reads for each library, for a total of approximately 380 million reads. After removing adapters and filtering out low quality sequences, 123 million clean 18–25 nt reads were counted, representing 20 million unique sequences. The majority of the sRNAs were 21–24 nt in size (94%), with 21 and 24 nt being the most frequently represented for all reads and unique reads (without reads redundancy), respectively (Figure 1a). These results were consistent with the representative size range of Dicer-like (DCL) cleavage products [40] and with the typical small RNA distribution of higher plants (e.g., in *At*, *Medicago truncatula* and *Paeonia suffruticosa* [41,42,43,44], supporting the reliability of the Illumina sequencing data for these sRNA libraries.

### 3.2. Identification of Conserved and Non-Conserved miRNAs

We predicted a total of 136 miRNAs and their hairpin precursors. Pre-miRNA lengths ranged from 76 to 400 nt and presented a high minimum folding energy index (MFEI) between 0.74 and 4.23. miRNA predictions were predominantly 21- (57%) and 24-nt long (20%) (Figure 1b) and initiated mainly with a 5′ U (64%) or A (22%) (Figure 1c). Canonical 21-nt miRNAs initiated mostly with a 5′ U (78%), whereas the majority of long 24-nt miRNAs started with a 5′ A (67%).

Among the 136 miRNA predictions, 102 (75%) presented alignments with known miRNAs from miRBase belonging to 28 distinct families (+11 that were not assigned to a miRNA family) (Table S1). The three most represented families were miR156, miR159 and miR166, with 12, 10 and 13 miRNAs, respectively. Seven of the 28 families represented in our dataset (miR156, miR159, miR160, miR166, miR171_1, miR390, and miR396) are highly conserved among plants. Indeed, they are represented in each plant lineage, from mosses to higher plants, for which miRNAs have been deposited into miRBase. The miRNAs belonging to these conserved families were represented by 5 million reads on average (Additional File S3). On the contrary, several miRNAs (including families miR169_6, miR862_2, miR1507, miR5037 and miR5770) that were retrieved only in Fabaceae species were represented by a much smaller number of reads, on average 13,336 (mean comparison using Mann-Whitney test: *p* = 0.001892). These results are consistent with previous studies indicating that conserved miRNAs are highly expressed and that most have several family members, whereas non-conserved ones are often weakly expressed and encoded by single loci [45].

The predicted miRNAs were also compared with the set of 68 miRNAs identified in pea developing seeds by Kreplak et al. [22]. Among the 68 miRNAs expressed in seeds, 47 were found in our analysis, indicating they are expressed in both seeds and aerial parts.

### 3.3. miRNAs Involved in Cold Response

**Figure 2:**
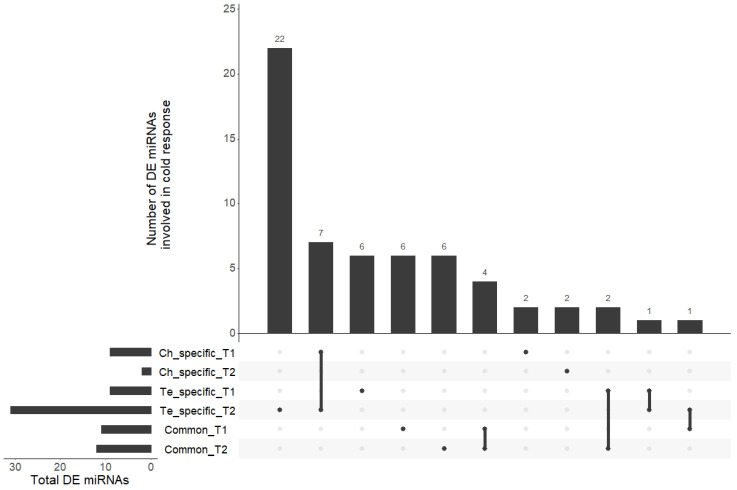
UpSet plot of the six lists of differentially abundant miRNAs. All intersections of the six sets of miRNAs are sorted by size. Dark circles in the matrix indicate sets that are part of each intersection.

Based on a comparison between the samples subjected to a cold acclimation treatment (4.5 °C; LT) or not (N) before applying frost, the differential analysis allowed the identification of 59 miRNAs involved in cold response (43.3% of the miRNAs) (Tables S2–S7, Additional File S4). Among them, 40 miRNAs presented a differential expression (LT vs. N) in only one of the two genotypes (i.e., genotype-specific responses) at T1 and/or T2, 16 presented a differential expression in the two genotypes (common responses) at T1 and/or T2, and 3 were found to be related to both specific and common responses at different time points (Figure 2).

Among miRNAs presenting a modulation of expression only in Ch, the miR159 family gathered 27% of the differentially abundant miRNAs. Four miRNA families were equally represented within Te-specific miRNAs: members of the miR164 and miR167_1 families were up-regulated at T1 and T2, a member of the miR159 family was down-regulated at T1 and up-regulated at T2, and a member of miR156 was up-regulated at T1 and down-regulated at T2. Among miRNAs related to the common response of the two genotypes, a member of the miR156 family was up-regulated at T1 and down-regulated at T2, whereas one of the miR166 family was up-regulated at T2.

### 3.4. Identification of miRNA Targets

**Figure 3:**
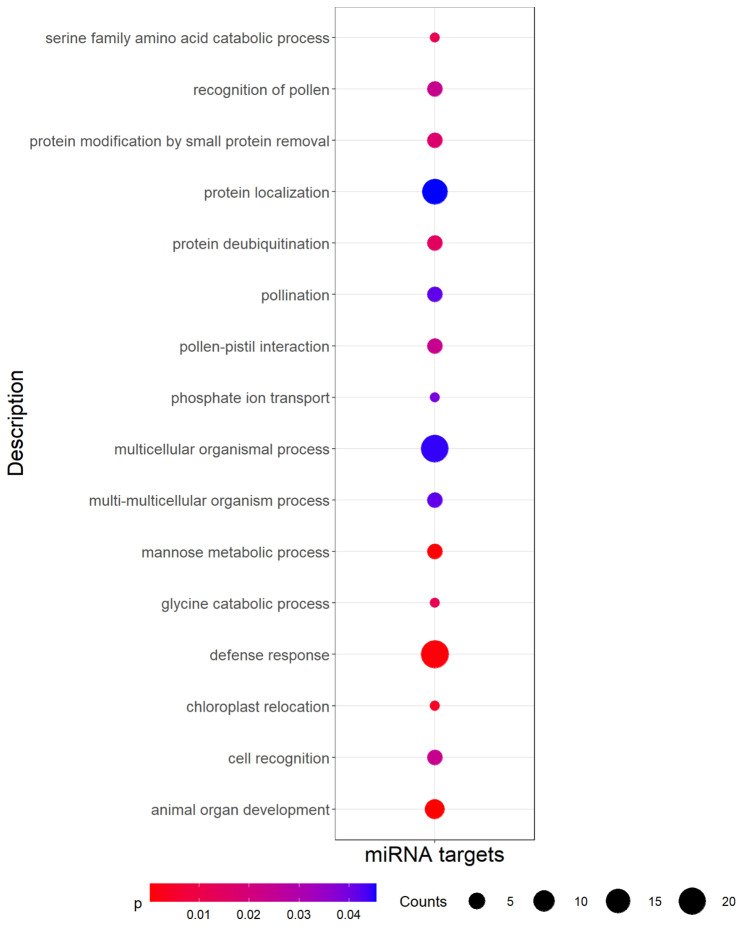
Significantly (*p*-value < 0.05) enriched Gene Ontology (GO) biological process terms among miRNA targets. Counts indicate the number of targets annotated with each GO term, and dots are colored by *p*-value.

We then predicted putative target genes for the 136 predicted miRNAs by looking for sequence complementarity with the coding sequences extracted from the pea genome annotation [22]. Among the 136 previously selected miRNAs, 106 (77.9%) were found to have putative targets (1789 transcripts representing 1253 distinct genes, Table S8). A Gene Ontology (GO) analysis performed on the set of target genes pointed out an enrichment of biological processes (BP) related to development and defense (Figure 3).

### 3.5. Differentially Expressed Genes in Response to Cold

The recent release of a pea reference genome (cv Cameor, [21]) enabled us to perform a new assessment of gene expression using RNA-seq data previously produced by Bahrman et al. [11] thanks to a pipeline similar to that used for the miRNA-seq data. This updated gene expression quantification facilitated comparisons and integration of RNA-seq and miRNA-seq results. Illumina sequencing of the twenty-four mRNA libraries previously produced by Bahrman et al. [11] yielded a total of 420 million pairs of reads. After filtering out low quality reads, more than 99.99% of these were aligned to the Cameor genome sequence. 393 million (94.3%) pairs could be aligned to the genome assembly, including 358 million with a single genomic location. 94% and 1% of these alignments corresponded to the coding and non-coding strands of annotated genes, respectively, and the remaining 5% were located outside any annotated gene.

**Figure 4:**
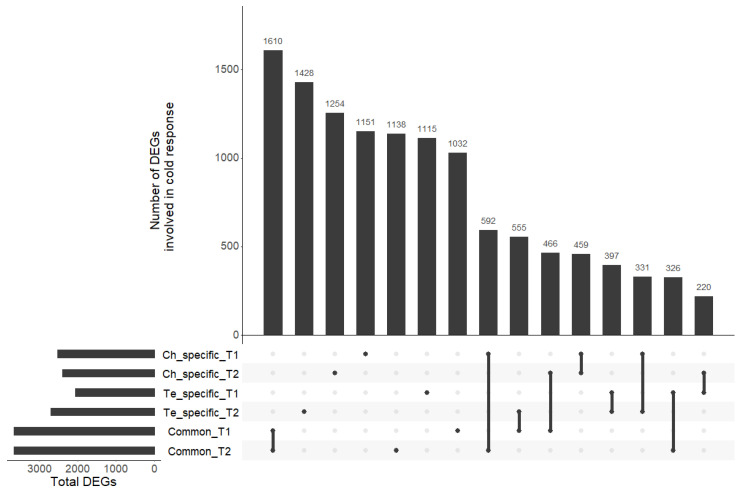
UpSet plot of the six sets of DEGs. All intersections of the six sets are sorted by size. Dark circles in the matrix indicate sets that are part of each intersection.

The transcriptomic responses of the two contrasted pea lines (Ch, frost tolerant; Te, frost susceptible) subjected to a cold acclimation treatment (4.5 °C; LT) or not (N) before applying frost was studied after different periods of cold exposure T1 and T2 [11]. For both periods, we identified four sets of DEGs: (1) between LT and N in Ch at T1 (Table S9); (2) between LT and N in Ch at T2 (Table S10); (3) between LT and N in Te at T1 (Table S11); and (4) between LT and N in Te at T2 (Table S12). These sets were then used to pinpoint Ch and Te-specific cold responsive genes (Tables S9–S12) and genes involved in cold response common to both genotypes (Tables S13 and S14). Among the various comparisons considered, we identified between two and four thousand differentially expressed genes under cold stress (Table 1). A total of 3663 and 3666 DEGs at T1 and T2, respectively, were involved in cold response in Ch and Te. A small number of genes was found to be involved in cold response at T1 and T2 in both genotypes but with discordant differential expressions. Some DEGs were found to have a consistent response regardless of the cold exposure period: 459 DEGs for the Ch-specific response, 397 DEGs for the Te-specific response, and 1610 DEGs for the common response to cold. We also observed cases of offsets in cold response between the two genotypes: for example, 220 DEGs in Te at T1 were also differentially expressed in Ch at T2, and 331 DEGs in Ch at T1 were differently expressed in Te at T2 (Figure 4).

**Figure 5:**
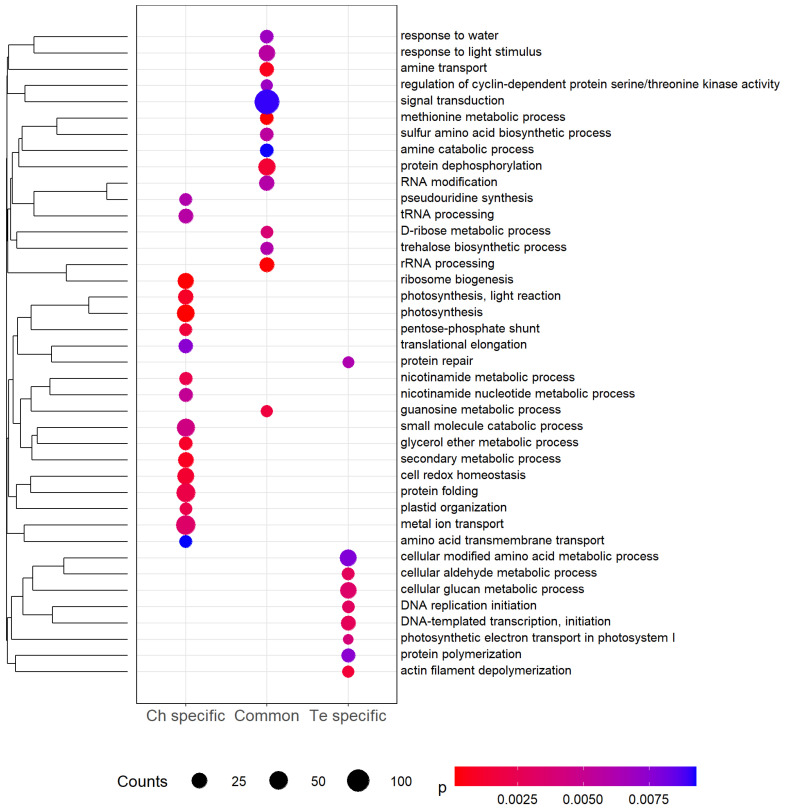
Significantly (*p*-value < 0.01) enriched Gene Ontology (GO) biological process terms among differentially expressed genes (DEGs) specific to Ch, Te, and common to the two genotypes. Counts indicate the number of DEGs annotated with each GO term, and dots are colored by *p*-value. GO terms are clustered using hierarchical clustering (complete linkage) using GOGO semantic similarity [38] between terms.

To investigate the function of cold-responsive DEGs, a GO enrichment analysis was performed on three separate gene lists (Figure 5, Tables S15–S17). To constitute the three lists, we first combined the results for the two different periods of cold exposure (T1 and T2); we then focused on Te-specific, Ch-specific, and common concordant cold-responsive genes. Signal transduction, protein dephosphorylation and response to light stimulus were the most enriched biological processes for DEGs common to both genotypes. For Ch-specific DEGs, photosynthesis, protein folding and metal ion transport were most highly enriched, while cellular modified amino acid and cellular glucan metabolic processes were most highly enriched for Te-specific DEGs. Based on a *p*-value significance threshold of 1%, no biological processes were found to be enriched across all three lists.

### 3.6. miRNAs and Target mRNAs with Discordant Expression Profiles

**Figure 6:**
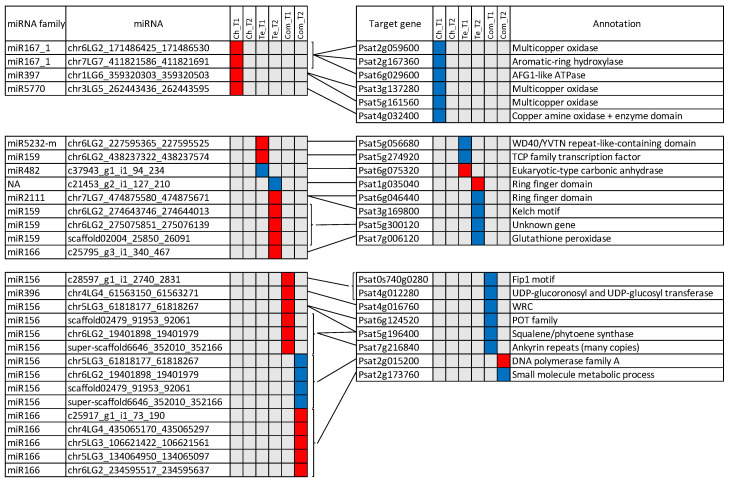
Discordant patterns of significant differential expression for miRNAs and their putative mRNA targets. Ch, Te and Com, respectively represent Ch-specific, Te-specific and common differential cold-response. Down- and up-regulated mRNAs and miRNAs are shown in blue and red, respectively. Lines joining miRNAs and mRNAs represent putative miRNA-target relationships.

Among the 1253 miRNA target genes identified, 252 were included in the lists of DEGs. In total, 84 miRNA/target DEG pairs were identified, with 39 of them presenting discordant expression patterns (Figure 6). For Ch-specific miRNA/mRNA pairs at T1, four miRNAs were up-regulated and six of their targets were down-regulated, including three multicopper oxidase. No miRNA/mRNA pairs were identified for Ch-specific genes and miRNAs involved in cold response at T2. For Te-specific cold response at T1, two miRNAs were up regulated, one was down regulated. At T2, six pairs including five target genes were found. In this case, 15 pairs of miRNAs/mRNAs were identified with a cold response common to the two pea genotypes. Interestingly, four miRNAs were up-regulated at T1 but down-regulated at T2 with a unique and different target DEG at T2, suggesting they have different targets at different time points.

## 4. Discussion

### 4.1. Metabolic Responses to Cold in Pea

The GO enrichment analysis we present here provides useful insight into the pea response to cold stress (Figure 5). For common differential cold-response in gene expression, signal transduction and protein dephosphorylation are the most highly enriched biological processes. Signal transduction and protein dephosphorylation have a key role in plant responses to environmental stresses such as drought, high salinity, cold, and pathogen attack. Kinase proteins are known as a key feature of signal transduction [46]. In this study, 177 DEGs (4.83%) and 199 DEGs (5.43%) encode protein kinases at T1 and T2, respectively, and 75 of these DEGs are common between T1 and T2. In the common differential cold-response, trehalose biosynthetic process is also a significantly enriched term. Trehalose is known as a membrane stabilizer and an inhibitor of membrane fusion [47]. For example, seven genes involved in trehalose synthesis are reported to be more expressed in *At* under cold exposure [48,49]. Polyamines can also stabilize the membranes and inhibit protein and mRNA denaturation. In the common differential cold response, several biological processes are linked to polyamines: amine catabolic process, amine transport, methionine metabolic process and sulfur amino acid biosynthetic process. Methionine and cysteine are substrates for the synthesis of various polyamines with important roles in stress tolerance, the most prominent being putrescine, spermidine and spermine [50]. Comparing two chickpea (*Cicer arietinum*) genotypes, higher levels of putrescine, spermidine and spermine were observed after six days of cold stress at 4 °C [51].

Metal ion transport, which appears in Ch-specific cold response (Figure 5), could be linked to the photosynthesis changes during cold stress. Fe, Cu, and Mn readily change their oxidative state, which enables these transition metals to participate in vital cellular processes such as photosynthesis or the electron transport chain [52]. In Ch, a higher inherent photosynthetic potential at the beginning of the cold exposure, combined with an early ability to start metabolic processes aimed at maintaining the photosynthetic capacity, has already been described [9]. This ability to start metabolic processes in Ch is also observed via other biological processes: secondary metabolic process, glycerol ether metabolic process or protein folding. Protein folding in vivo is mediated by an array of proteins that act as molecular chaperones. Molecular chaperones share the property that they bind substrate proteins that are in unstable, non-native structural states [53]. Among the chaperone proteins, heat shock proteins (HSPs) are known to enhance stress tolerance such as temperature shift. In *At*, four genes encoding HSP70 proteins are up-regulated after 48 h at 4 °C [54]. In *Nt*, 10 *HSP70* genes out of 61 are up-regulated during cold treatment [55]. In Ch, three *HSP70* genes are up-regulated: *Psat7g103280* and *Psat3g071280* at T1, *Psat7g103280* and *Psat1g222760* at T2.

Several biological processes involved in Ch-specific cold response and common differential cold-response are linked to Te-specific cold response biological processes. Similar to trehalose, glucans stabilize the membranes and inhibit membrane fusion [56]. As in Ch, cold stress induces photosynthetic changes in Te with a modification of the electron transport in photosystem I. In Te, the cytoskeleton undergoes modification during exposure to low temperature with a significant enrichment of the GO term actin filament depolymerization. It has been shown that the rearrangement of the cytoskeleton is also linked to the induction of genes related to cold acclimation in *At* cell suspensions and *Brassica napus* leaves [57,58]. In *Nt* cell suspensions at 0 °C, the microtubules and filaments form bundles [59] and, in *Vitis rupestris* cell suspensions at 0 °C, microtubules totally disassemble [60].

### 4.2. Regulation of the Cold Response by miRNAs in Pea

Eleven miRNA families were represented in the 39 miRNA/target DEG pairs identified in this study, including miR156, miR159, miR166, miR167_1, miR396, miR397, miR482, miR2111, miR5770, miR5232 and a miRNA sequence which was not annotated. Their corresponding putative target genes provide a first insight into the main miRNA regulatory pathways involved in the pea response to cold.

#### 4.2.1. Antioxidative Processes Are Predominantly Highlighted in Both Genotypes

Eight of the 22 miRNA targets differentially expressed in this study are annotated as genes potentially implied in antioxidative processes under abiotic stresses in plants. In our study, they appeared to be either commonly regulated in both genotypes or regulated in the sensitive genotype Te.

A first group of 3 genes, namely *Psat4g012280*, *Psat6g124520* and *Psat5g056680*, respectively, annotated as UDP-glucoronosyl and UDP-glucosyl transferase, POT family and WD40/YVTN repeat-like-containing domain, are known to participate in the synthesis, regulation or transport of flavonoids. Flavonoids, including anthocyanins, play an important role in scavenging reactive oxygen species (ROS) implicated in many biotic and abiotic stresses. In *At*, for example, the expression of a citrus *UDP glucosyl transferase* gene enhanced the accumulation of anthocyanins and flavonoids, which was related to a better antioxidant potential under high light stress [61]. In tea leaves (*Camellia sinensis* L.), the *CsUGT75C1* gene, encoding UDP-glucose:anthocyanin 5-O-glucosyl transferase, was up-regulated in response to cold, along with an increase of the anthocyanin content [62]. In rice, a POT protein was mentioned as a component of the regulation of flavonoid metabolism during pollen intine formation [63]. In pea, many genetic loci are known to affect the synthesis of anthocyanins and were described for their role in the pigmentation of flowers and other organs [64,65]. Only three of these loci have been identified until now [66,67] and their sequence allowed us to note that none of them corresponded to the target genes mentioned here.

The gene *Psat5g196400* is annotated as squalene/phytoene synthase, an enzyme which regulates the first step of the carotenoid biosynthetic pathway in plants. Similar to flavonoids, the carotenoid pigments are known to protect photosynthesis from ROS under various environmental stresses [68]. Phytoene synthase expression was shown to be regulated under abiotic stresses such as heat and drought stress in wheat [69] and heat stress in maize [70]. In these species, the allelic variation of *PSY* genes has been explored to breed for enhanced carotenoids levels under stresses.

In addition to the regulation of pigments, four other genes are potentially implied in ROS scavenging. They are *Psat7g006120*, *Psat7g216840*, *Psat6g046440* and *Psat1g035040*, respectively, annotated as glutathione peroxydase, ankyrin repeats and ring finger domain for the last two. Glutathione peroxidase participates in ROS enzymatic scavenging systems in plants by reducing H_2_O_2_ and organic and lipid hydroperoxides [71]. In winter rapeseed, for instance, the coordinated level of superoxyde dismutase, ascorbate peroxydase and glutathione peroxydase activities in cold acclimated young leaves of the cultivar Hansen was shown to play an essential role in order to avoid oxidative damage during cold exposure [72]. Plant ankyrin repeat (ANK) proteins are involved in various important biological processes, including response to biotic and abiotic stresses [73]. For example, the *GmANK114* gene was overexpressed in soybean hairy roots in response to drought or salt stress, and lower ROS contents were also observed comparatively to control plants. The ring finger domain proteins are a large family, among which the ring-type E3-ubiquitin ligases have received a particular attention due to their potential impact in plant development, hormonal control and defense against biotic and abiotic stresses [74]. Regarding the response to cold stress, Fang et al. [75] functionally characterized the rice *OsSRFP1* gene, coding the stress-related ring finger protein 1. They evidenced the increased cold tolerance of *OsSRFP1* knock-down rice plants, associated with higher amounts of free proline and activities of antioxidant enzymes.

#### 4.2.2. Three Conserved miRNA Families Are Highly Represented in the Pea Response to Cold and Could Regulate the Response to Multiple Stresses as Well as Plant Morphology and Phenology

miR156, miR166 and miR159, which are miRNAs conserved among plants, are the most strongly represented miRNAs in the present study. Five among the eight genes discussed above were regulated by members of the miR156 or miR166 families, as were almost all the genes commonly regulated by Ch and Te. The differential expression of miR156 and miR166 under low temperature conditions has been shown in various plant species [18] in organs of plantlets which are comparable to those studied here for pea. In *Brassica rapa*, for instance, miR156 and miR166 appeared among the 11 miRNA families whose members showed differential expression patterns under freezing stress (−4 °C), compared to control [76]. In *Triticum aestivum*, miR156, miR166 and miR159 were differentially expressed between cold (0 °C) and control [77]. In *Medicago sativa*, which such as *Ps* belongs to the Fabaceae family, the same three miRNAs were represented among the 27 miRNA families differentially expressed under cold (4 °C) and freezing (−8 °C) conditions [78].

Among cold responsive miRNAs, miR156 is also known to play an important part in plant morphology and in transition from the vegetative to the reproductive phase. Moreover, a coordinating role of miR156 in the relationship between plant development and abiotic stress tolerance has been demonstrated in *At* under salt and drought signals [79]. This previous study evidenced that the expression level of miR156 regulated, in a coordinated way, downstream genes implied in floral transition and in anthocyanin biosynthesis. It is interesting to notice that, in the present study, miR156 simultaneously regulated *Psat0s740g0280*, annotated as Fip1 motif, and *Psat4g012280* discussed above, annotated as UDP-glucoronosyl and UDP-glucosyl transferase. In *At*, *FIP1* is thought to mediate alternative polyadenylation and thus to contribute to regulation of the transcriptome [80,81]. The *fip1-2* mutant of *At* exhibited multiple phenotypes affecting plant height, leaf size, flowering time and response to Cadmium and salt stress [81]. Additionally, as stated above, UDP glucosyl transferase is implied in the production of anthocyanins under cold conditions [62], which could be related to ROS scavenging. Thus, even if the miRNA-target modules vary among species, it would be of great interest to further study in pea the potential co-regulation of plant morphology, phenology and response to cold by miR156.

Lastly, in addition to their response to cold, there is also evidence of the induction of miR156, miR159 and miR166 by multiple stresses, including biotic stresses. For instance, in wheat, these three miRNAs were induced in response to cold [77], but also in response to powdery mildew [82]. In pea, the co-regulation by miRNAs of the cold and biotic stress responses merits additional exploration, as the question of a common genetic determinism of frost tolerance and *Ascochyta* resistance also arises from the colocalization of main QTLs (quantitative trait loci) for both traits [83].

#### 4.2.3. Laccases Are Specifically Involved in Cold Adaptation of the Tolerant Genotype Ch

Six pairs of miRNAs/mRNAs with discordant expression patterns were identified at T1 for the frost tolerant pea accession Ch. Half of the miRNA targets were annotated as multicopper oxidases, which are an enzyme superfamily widely distributed in avian, bacteria, fungi, insects, mammalians and plants [84]. Using InterProScan [85], *Psat2g059600*, *Psat3g137280* and *Psat5g161560* were identified more precisely as laccases. In the present study, they were regulated by members of miR167, miR397 and miR5770 families. The miR397-laccase module has been particularly reported as an actor of plant response to cold in various plant species. As laccases are involved in lignin polymerization, one hypothesis is that miR397 directly regulates cold tolerance via reduction of lignin content in cell wall, thereby increasing cell wall permeability and elasticity, which can be a mechanical advantage under cold and freezing stress [18]. A second hypothesis, based on an indirect role of miR397 and miR397 targets in the *CBF* (*C-repeat Binding Factor*) regulon, an important regulatory pathway of the cold response, was also evoked by Dong and Pei [19]. These authors showed that transgenic plants overexpressing miR397a exhibited an improved tolerance to chilling (4 °C) and freezing (−1 °C) stresses and that overexpression of miR397a was associated with enhanced transcript levels of cold-regulated *CBF* genes and downstream *COR* (*Cold Responsive*) genes. They thus hypothesized that the transcription of *CBF* and *COR* genes was regulated by the miR397 target genes (3 laccases and a casein kinase β subunit 3 in this study). This complicated regulation would require further study in pea where *CBF* genes have been identified among candidate genes underlying a major QTL for frost tolerance located on linkage group VI [8]. Interestingly, the favorable allele at this QTL is contributed by the frost tolerant genotype Ch, which also showed a specific regulation of laccases in response to cold in this study. Lastly, it is interesting to consider that miR397 has also been shown to regulate the response to biotic stress, as in *Malus hupehensis*, a *Malus* rootstock species, where transient overexpression of miR397b in leaves triggered an increased sensitivity to *Botryosphaeria dothidea*, owing to the reduced Laccase7 and lignin contents [86]. As such, the miR397-laccase module should be studied in pea as a potential determinant of co-regulation of cold and biotic stress responses.

## 5. Conclusions

This study contributes new insight on the mechanisms implemented in pea during cold stress response by elucidating miRNA-mediated regulations. Our functional investigation of miRNA-target modules particularly highlighted antioxidative mechanisms in both a tolerant and a sensitive pea genotype. The tolerant line moreover showed specific expression of the miR397-laccase module, which could have either a direct role in cell wall remodeling under cold exposure or an indirect role in cold acclimation via the *CBF* regulon. Finally, the expression of miRNA families which are conserved among plant species, such as miR156, miR159, miR166 and miR397, suggests that these miRNAs could participate in the co-regulation of the responses to cold and biotic stresses in pea, as well as in the regulation of plant morphology and phenology under low temperature.

## Figures and Tables

**Table 1:** Summary of DEGs identified in Champagne (Ch) and Térèse (Te) in response to cold in time periods T1 and T2 (adjusted *p*-values < 5%).

List of DEGs		DEGs at T1	DEGs at T2
Te specific cold responsive genes		(−) 1171 (+) 887	(−) 1569 (+) 1142
Ch specific cold responsive genes		(−) 1332 (+) 1201	(−) 1162 (+) 1237
Te and Ch common cold responsive genes	Concordant genes	(−) 1719 (+) 1921	(−) 2000 (+) 1613
	Discordant genes	12 (+) Te & (−) Ch 11 (−) Te & (+) Ch	19 (+) Te & (−) Ch 34 (−) Te & (+) Ch

(−) Down regulated genes, (+) Up regulated genes.

## Data Availability

Raw sRNA-seq data were transferred to the NCBI-SRA database, in the BioProject PRJNA543764.

## Supplementary Materials

The following supporting information can be downloaded at: https://www.mdpi.com/article/10.3390/genes13071119/s1, Additional File S1 contains a schematic representation of the study design. Additional File S2 comprises Table S1 (miRNAs identified in this study), Table S2 (Ch specific differentially expressed miRNAs at T1), Table S3 (Ch specific differentially expressed miRNAs at T2), Table S4 (Te specific differentially expressed miRNAs at T1), Table S5 (Te specific differentially expressed miRNAs at T2), Table S6 (Common miRNAs differentially expressed at T1), Table S7 (Common miRNAs differentially expressed at T2), Table S8 (Identification of miRNA targets), Table S9 (Ch specific differentially expressed genes at T1), Table S10 (Ch specific differentially expressed genes at T2), Table S11 (Te specific differentially expressed genes at T1), Table S12 (Te specific differentially expressed genes at T2), Table S13 (Common genes differentially expressed at T1), Table S14 (Common genes differentially expressed at T1), Table S15 (GO terms enrichment for the Ch specific differentially expressed genes), Table S16 (GO terms enrichment for the Te specific differentially expressed genes) and Table S17 (GO terms enrichment for the common genes differentially expressed). Additional File S3 represents the expression of conserved and Fabaceae-specific miRNAs. Additional File S4 represents principal component analyses of miRNA (A) and mRNA (B) samples.

## References

[1] Ferreira H., Vasconcelos M., Gil A.M., Pinto E. (2021). Benefits of pulse consumption on metabolism and health: A systematic review of randomized controlled trials. Crit. Rev. Food Sci. Nutr..

[2] Vaz Patto M.C., Amarowicz R., Aryee A.N.A., Boye J.I., Chung H.-J., Martín-Cabrejas M.A., Domoney C. (2015). Achievements and Challenges in Improving the Nutritional Quality of Food Legumes. Crit. Rev. Plant Sci..

[3] Jeuffroy M.H., Baranger E., Carrouee B., de Chezelles E., Gosme M., Henault C., Schneider A., Cellier P. (2013). Nitrous oxide emissions from crop rotations including wheat, oilseed rape and dry peas. Biogeosciences.

[4] Stagnari F., Maggio A., Galieni A., Pisante M. (2017). Multiple benefits of legumes for agriculture sustainability: An overview. Chem. Biol. Technol. Agric..

[5] Voisin A.S., Gueguen J., Huyghe C., Jeuffroy M.H., Magrini M.B., Meynard J.M., Mougel C., Pellerin S., Pelzer E. (2014). Legumes for feed, food, biomaterials and bioenergy in Europe: A review. Agron. Sustain. Dev..

[6] Duc G., Agrama H., Bao S.Y., Berger J., Bourion V., De Ron A.M., Gowda C.L.L., Mikic A., Millot D., Singh K.B. (2015). Breeding Annual Grain Legumes for Sustainable Agriculture: New Methods to Approach Complex Traits and Target New Cultivar Ideotypes. Crit. Rev. Plant Sci..

[7] Lejeune-Hénaut I., Hanocq E., Béthencourt L., Fontaine V., Delbreil B., Morin J., Petit A., Devaux R., Boilleau M., Stempniak J.J. (2008). The flowering locus Hr colocalizes with a major QTL affecting winter frost tolerance in *Pisum sativum* L.. Theor. Appl. Genet..

[8] Beji S., Fontaine V., Devaux R., Thomas M., Negro S.S., Bahrman N., Siol M., Aubert G., Burstin J., Hilbert J.-L. (2020). Genome-wide association study identifies favorable SNP alleles and candidate genes for frost tolerance in pea. BMC Genom..

[9] Grimaud F., Renaut J., Dumont E., Sergeant K., Lucau-Danila A., Blervacq A.-S., Sellier H., Bahrman N., Lejeune-Hénaut I., Delbreil B. (2013). Exploring chloroplastic changes related to chilling and freezing tolerance during cold acclimation of pea (*Pisum sativum* L.). J. Proteom..

[10] Legrand S., Marque G., Blassiau C., Bluteau A., Canoy A.-S., Fontaine V., Jaminon O., Bahrman N., Mautord J., Morin J. (2013). Combining gene expression and genetic analyses to identify candidate genes involved in cold responses in pea. J. Plant Physiol..

[11] Bahrman N., Hascoët E., Jaminon O., Dépta F., Hû J.-F., Bouchez O., Lejeune-Hénaut I., Delbreil B., Legrand S. (2019). Identification of Genes Differentially Expressed in Response to Cold in *Pisum sativum* Using RNA Sequencing Analyses. Plants.

[12] Rogers K., Chen X.M. (2013). Biogenesis, Turnover, and Mode of Action of Plant MicroRNAs. Plant Cell.

[13] Zhou X.R., Khare T., Kumar V. (2020). Recent trends and advances in identification and functional characterization of plant miRNAs. Acta Physiol. Plant..

[14] Chaudhary S., Grover A., Sharma P.C. (2021). MicroRNAs: Potential Targets for Developing Stress-Tolerant Crops. Life.

[15] Bhogireddy S., Mangrauthia S.K., Kumar R., Pandey A.K., Singh S., Jain A., Budak H., Varshney R.K., Kudapa H. (2021). Regulatory non-coding RNAs: A new frontier in regulation of plant biology. Funct. Integr. Genom..

[16] Pagano L., Rossi R., Paesano L., Marmiroli N., Marmiroli M. (2021). miRNA regulation and stress adaptation in plants. Environ. Exp. Bot..

[17] Liu Q., Yan S.J., Yang T.F., Zhang S.H., Chen Y.Q., Liu B. (2017). Small RNAs in regulating temperature stress response in plants. J. Integr. Plant Biol..

[18] Megha S., Basu U., Kav N.N.V. (2018). Regulation of low temperature stress in plants by microRNAs. Plant Cell Environ..

[19] Dong C.H., Pei H.X. (2014). Over-expression of miR397 improves plant tolerance to cold stress in *Arabidopsis thaliana*. J. Plant Biol..

[20] Chen L., Luan Y.S., Zhai J.M. (2015). Sp-miR396a-5p acts as a stress-responsive genes regulator by conferring tolerance to abiotic stresses and susceptibility to *Phytophthora nicotianae* infection in transgenic tobacco. Plant Cell Rep..

[21] Zhang X.N., Wang W., Wang M., Zhang H.Y., Liu J.H. (2016). The miR396b of Poncirus trifoliata Functions in Cold Tolerance by Regulating ACC Oxidase Gene Expression and Modulating Ethylene-Polyamine Homeostasis. Plant Cell Physiol..

[22] Kreplak J., Madoui M.A., Capal P., Novak P., Labadie K., Aubert G., Bayer P.E., Gali K.K., Syme R.A., Main D. (2019). A reference genome for pea provides insight into legume genome evolution. Nat. Genet..

[23] BioProject. PRJNA543764. https://www.ncbi.nlm.nih.gov/bioproject/543764.

[24] Martin M. (2011). Cutadapt removes adapter sequences from high-throughput sequencing reads. EMBnet J..

[25] Schmieder R., Edwards R. (2011). Quality control and preprocessing of metagenomic datasets. Bioinformatics.

[26] FastQC. http://www.bioinformatics.babraham.ac.uk/projects/fastqc/.

[27] Langmead B. (2010). Aligning Short Sequencing Reads with Bowtie. Curr. Protoc. Bioinform..

[28] Guigon I., Legrand S., Berthelot J.F., Bini S., Lanselle D., Benmounah M., Touzet H. (2019). miRkwood: A tool for the reliable identification of microRNAs in plant genomes. BMC Genom..

[29] Kozomara A., Birgaoanu M., Griffiths-Jones S. (2019). miRBase: From microRNA sequences to function. Nucleic Acids Res..

[30] Slater G.S., Birney E. (2005). Automated generation of heuristics for biological sequence comparison. BMC Bioinform..

[31] Fahlgren N., Carrington J.C. (2010). MiRNA Target Prediction in Plants. Methods Mol. Biol. Clifton NJ.

[32] Kim D., Landmead B., Salzberg S.L. (2015). HISAT: A fast spliced aligner with low memory requirements. Nat. Methods.

[33] Cock P.J.A., Antao T., Chang J.T., Chapman B.A., Cox C.J., Dalke A., Friedberg I., Hamelryck T., Kauff F., Wilczynski B. (2009). Biopython: Freely available Python tools for computational molecular biology and bioinformatics. Bioinformatics.

[34] Anders S., Pyl P.T., Huber W. (2015). HTSeq-a Python framework to work with high-throughput sequencing data. Bioinformatics.

[35] Pysam. https://github.com/pysam-developers/pysam.

[36] Robinson M.D., McCarthy D.J., Smyth G.K. (2010). edgeR: A Bioconductor package for differential expression analysis of digital gene expression data. Bioinformatics.

[37] Robinson M.D., Oshlack A. (2010). A scaling normalization method for differential expression analysis of RNA-seq data. Genome Biol..

[38] Alexa A., Rahnenfuhrer J. TopGO: Enrichment Analysis for Gene Ontology, R Package version 2.42.0; Bioconductor 2020. https://bioconductor.org/packages/release/bioc/html/topGO.html.

[39] Zhao C.G., Wang Z. (2018). GOGO: An improved algorithm to measure the semantic similarity between gene ontology terms. Sci. Rep..

[40] Henderson I.R., Zhang X.Y., Lu C., Johnson L., Meyers B.C., Green P.J., Jacobsen S.E. (2006). Dissecting *Arabidopsis thaliana* DICER function in small RNA processing, gene silencing and DNA methylation patterning. Nat. Genet..

[41] Moldovan D., Spriggs A., Yang J., Pogson B.J., Dennis E.S., Wilson I.W. (2010). Hypoxia-responsive microRNAs and trans-acting small interfering RNAs in Arabidopsis. J. Exp. Bot..

[42] Chen L., Wang T.Z., Zhao M.G., Tian Q.Y., Zhang W.H. (2012). Identification of aluminum-responsive microRNAs in *Medicago truncatula* by genome-wide high-throughput sequencing. Planta.

[43] Liu Y.X., Wang M., Wang X.J. (2014). Endogenous small RNA clusters in plants. Genom. Proteom. Bioinform..

[44] Zhang Y.X., Wang Y.Y., Gao X.K., Liu C.Y., Gai S.P. (2018). Identification and characterization of microRNAs in tree peony during chilling induced dormancy release by high-throughput sequencing. Sci. Rep..

[45] Cuperus J.T., Fahlgren N., Carrington J.C. (2011). Evolution and Functional Diversification of MIRNA Genes. Plant Cell.

[46] Zhu J.K. (2016). Abiotic Stress Signaling and Responses in Plants. Cell.

[47] Wolfe J., Bryant G. (1999). Freezing, drying, and/or vitrification of membrane-solute-water systems. Cryobiology.

[48] Iordachescu M., Imai R. (2008). Trehalose biosynthesis in response to abiotic stresses. J. Integr. Plant Biol..

[49] Kreps J.A., Wu Y.J., Chang H.S., Zhu T., Wang X., Harper J.F. (2002). Transcriptome changes for Arabidopsis in response to salt, osmotic, and cold stress. Plant Physiol..

[50] Zagorchev L., Seal C.E., Kranner I., Odjakova M. (2013). A Central Role for Thiols in Plant Tolerance to Abiotic Stress. Int. J. Mol. Sci..

[51] Amini S., Maali-Amiri R., Kazemi-Shahandashti S.S., Lopez-Gomez M., Sadeghzadeh B., Sobhani-Najafabadi A., Kariman K. (2021). Effect of cold stress on polyamine metabolism and antioxidant responses in chickpea. J. Plant Physiol..

[52] Bashir K., Ahmad Z., Kobayashi T., Seki M., Nishizawa N.K. (2021). Roles of subcellular metal homeostasis in crop improvement. J. Exp. Bot..

[53] Boston R.S., Viitanen P.V., Vierling E. (1996). Molecular chaperones and protein folding in plants. Plant Mol. Biol..

[54] Sung D.Y., Vierling E., Guy C.L. (2001). Comprehensive expression profile analysis of the Arabidopsis hsp70 gene family. Plant Physiol..

[55] Song Z.P., Pan F.L., Lou X.P., Wang D.B., Yang C., Zhang B.Q., Zhang H.Y. (2019). Genome-wide identification and characterization of Hsp70 gene family in *Nicotiana tabacum*. Mol. Biol. Rep..

[56] Ambroise V., Legay S., Guerriero G., Hausman J.-F., Cuypers A., Sergeant K. (2019). The Roots of Plant Frost Hardiness and Tolerance. Plant Cell Physiol..

[57] Orvar B.L., Sangwan V., Omann F., Dhindsa R.S. (2000). Early steps in cold sensing by plant cells: The role of actin cytoskeleton and membrane fluidity. Plant J..

[58] Sangwan V., Foulds I., Singh J., Dhindsa R.S. (2001). Cold-activation of *Brassica napus* BN115 promoter is mediated by structural changes in membranes and cytoskeleton, and requires Ca2+ influx. Plant J..

[59] Pokorna J., Schwarzerova K., Zelenkova S., Petrasek J., Janotova I., Capkova V., Opatrny Z. (2004). Sites of actin filament initiation and reorganization in cold-treated tobacco cells. Plant Cell Environ..

[60] Wang L.X., Sadeghnezhad E., Riemann M., Nick P. (2019). Microtubule dynamics modulate sensing during cold acclimation in grapevine suspension cells. Plant Sci..

[61] Rao M.J., Xu Y.T., Huang Y., Tang X.M., Deng X.X., Xu Q. (2019). Ectopic expression of citrus UDP-GLUCOSYL TRANSFERASE gene enhances anthocyanin and proanthocyanidins contents and confers high light tolerance in Arabidopsis. BMC Plant Biol..

[62] Shen J.Z., Zhang D.Y., Zhou L., Zhang X.Z., Liao J.R., Duan Y., Wen B., Ma Y.C., Wang Y.H., Fang W.P. (2019). Transcriptomic and metabolomic profiling of *Camellia sinensis* L. cv. ‘Suchazao’ exposed to temperature stresses reveals modification in protein synthesis and photosynthetic and anthocyanin biosynthetic pathways. Tree Physiol..

[63] Zhang Y.C., He R.R., Lian J.P., Zhou Y.F., Zhang F., Li Q.F., Yu Y., Feng Y.Z., Yang Y.W., Lei M.Q. (2020). OsmiR528 regulates rice-pollen intine formation by targeting an uclacyanin to influence flavonoid metabolismz. Proc. Natl. Acad. Sci. USA.

[64] Statham C.M., Crowden R.K., Harborne J.B. (1972). Biochemical genetics of pigmentation in *Pisum sativum*. Phytochemistry.

[65] Marx G.A., Weeden N.F., Muehlbauer F.J. (1989). A-2: A new locus controlling anthocyanin production in *Pisum*. Pisum Newsl..

[66] Hellens R.P., Moreau C., Lin-Wang K., Schwinn K.E., Thomson S.J., Fiers M.W.E.J., Frew T.J., Murray S.R., Hofer J.M.I., Jacobs J.M.E. (2010). Identification of Mendel’s White Flower Character. PLoS ONE.

[67] Moreau C., Ambrose M.J., Turner L., Hill L., Ellis T.H.N., Hofer J.M.I. (2012). The b Gene of Pea Encodes a Defective Flavonoid 3′,5′-Hydroxylase, and Confers Pink Flower Color. Plant Physiol..

[68] Demmig-Adams B., Adams W.W. (2002). Antioxidants in photosynthesis and human nutrition. Science.

[69] Flowerika, Alok A., Kumar J., Thakur N., Pandey A., Pandey A.K., Upadhyay S.K., Tiwari S. (2016). Characterization and Expression Analysis of Phytoene Synthase from Bread Wheat (*Triticum aestivum* L.). PLoS ONE.

[70] Li F.Q., Vallabhaneni R., Yu J., Rocheford T., Wurtzel E.T. (2008). The maize phytoene synthase gene family: Overlapping roles for carotenogenesis in endosperm, photomorphogenesis, and thermal stress tolerance. Plant Physiol..

[71] Gill S.S., Tuteja N. (2010). Reactive oxygen species and antioxidant machinery in abiotic stress tolerance in crop plants. Plant Physiol. Biochem..

[72] Dogru A., Cakirlar H. (2020). Effects of leaf age on chlorophyll fluorescence and antioxidant enzymes activity in winter rapeseed leaves under cold acclimation conditions. Braz. J. Bot..

[73] Vo K.T.X., Kim C.Y., Chandran A.K.N., Jung K.H., An G., Jeon J.S. (2015). Molecular insights into the function of ankyrin proteins in plants. J. Plant Biol..

[74] Chen L., Hellmann H. (2013). Plant E3 Ligases: Flexible Enzymes in a Sessile World. Mol. Plant.

[75] Fang H.M., Meng Q.L., Zhang H.S., Huang J. (2016). Knock-down of a RING finger gene confers cold tolerance. Bioengineered.

[76] Zeng X., Xu Y., Jiang J., Zhang F., Ma L., Wu D., Wang Y., Sun W. (2018). Identification of cold stress responsive microRNAs in two winter turnip rape (*Brassica rapa* L.) by high throughput sequencing. BMC Plant Biol..

[77] Song G., Zhang R., Zhang S., Li Y., Gao J., Han X., Chen M., Wang J., Li W., Li G. (2017). Response of microRNAs to cold treatment in the young spikes of common wheat. BMC Genom..

[78] Shu Y.J., Liu Y., Li W., Song L.L., Zhang J., Guo C.H. (2016). Genome-Wide Investigation of MicroRNAs and Their Targets in Response to Freezing Stress in *Medicago sativa* L., Based on High-Throughput Sequencing. G3 Genes Genom. Genet..

[79] Cui L.G., Shan J.X., Shi M., Gao J.P., Lin H.X. (2014). The miR156-SPL9-DFR pathway coordinates the relationship between development and abiotic stress tolerance in plants. Plant J..

[80] Forbes K.P., Addepalli B., Hunt A.G. (2006). An Arabidopsis Fip1 homolog interacts with RNA and provides conceptual links with a number of other polyadenylation factor subunits. J. Biol. Chem..

[81] Tellez-Robledo B., Manzano C., Saez A., Navarro-Neila S., Silva-Navas J., de Lorenzo L., Gonzalez-Garcia M.P., Toribio R., Hunt A.G., Baigorri R. (2019). The polyadenylation factor FIP1 is important for plant development and root responses to abiotic stresses. Plant J..

[82] Xin M.M., Wang Y., Yao Y.Y., Xie C.J., Peng H.R., Ni Z.F., Sun Q.X. (2010). Diverse set of microRNAs are responsive to powdery mildew infection and heat stress in wheat (*Triticum aestivum* L.). BMC Plant Biol..

[83] Boutet G., Lavaud C., Coyne C.J., Philippe D., Lejeune-Henaut I., Lesné A., Pilet-Nayel M.-L., Baranger A. Identification of regions in the pea genome controlling both stress resistance and developmental traits. Proceedings of the ICLGG 2019.

[84] McCaig B.C., Meagher R.B., Dean J.F.D. (2005). Gene structure and molecular analysis of the laccase-like multicopper oxidase (LMCO) gene family in *Arabidopsis thaliana*. Planta.

[85] Jones P., Binns D., Chang H.Y., Fraser M., Li W.Z., McAnulla C., McWilliam H., Maslen J., Mitchell A., Nuka G. (2014). InterProScan 5: Genome-scale protein function classification. Bioinformatics.

[86] Yu X.Y., Gong H.Y., Cao L.F., Hou Y.J., Qu S.C. (2020). MicroRNA397b negatively regulates resistance of *Malus hupehensis* to *Botryosphaeria dothidea* by modulating MhLAC7 involved in lignin biosynthesis. Plant Sci..

