# Supplementary material for: Integrated sRNA-seq and RNA-seq Analyses Reveal a microRNA Regulation Network Involved in Cold Response in Pisum sativum L
Source: Genes (Basel). 2022 Jun 22;13(7):1119. doi: 10.3390/genes13071119 (PMC9322779; doi:10.3390/genes13071119)
Supplement: Supplementary file 1 [file genes-13-01119-s001.zip › Additional file S1.pdf]

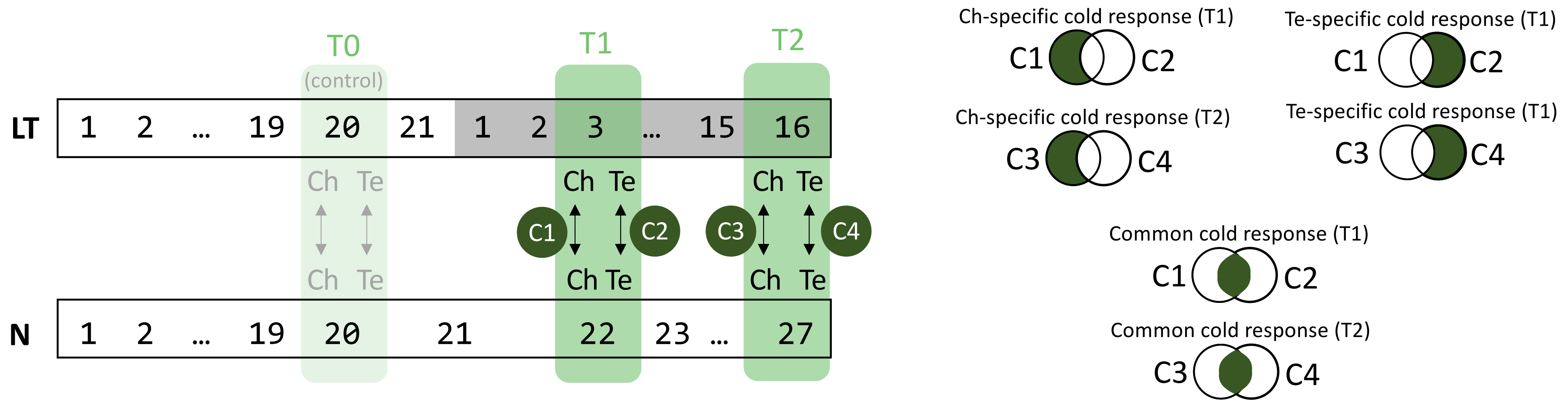

Additional file S1: Schematic representation of the study design

Champagne, and Térèse, were grown under contrasted temperature conditions within 2 experiments named LT (low temperature treatment: acclimation phase) and N (control: without acclimation). After an identical nursery period (shown on a white background) (21 days at 20°C day/14°C night), plants were exposed either to a low temperature treatment (shown on a grey background) (LT; 16 days at 8°C day/2°C night, i.e. a daily average temperature of 4.5°C), or to an extended nursery period (N; 6 more days at 20°C day/14°C night). During the LT or control N treatment, plants were sampled 3 times (T0, T1 and T2, in green). In both experiments, T1 corresponded to 386 degree.days, *i.e.* T0 + 36 degree.days and T2 corresponded to 472 degree.days *i.e.* T0 + 122 degree.days.

Contrasts to identify differentially expressed mRNAs or miRNAs between pairs of genotypes within an experiment are represented as arrows and labeled with dark green circles. Champagne-specific cold responsive mRNAs or miRNAs at T1 correspond to differentially expressed elements in contrast C1 but not in C2, while Térèse-specific cold responsive mRNAs or miRNAs at T1 correspond to differentially expressed elements in contrast C2 but not C1. Common (Champagne and Térèse) cold responsive mRNAs or miRNAs at T1 correspond to differentially expressed elements in both C1 and C2. Contrasts for T2 have similar interpretations. T0 (in light green) was treated as a control time point, and mRNAs or miRNAs with unexpected differential expression between the LT and N conditions at T0 were removed.
