# Supplementary material for: Integrated sRNA-seq and RNA-seq Analyses Reveal a microRNA Regulation Network Involved in Cold Response in Pisum sativum L
Source: Genes (Basel). 2022 Jun 22;13(7):1119. doi: 10.3390/genes13071119 (PMC9322779; doi:10.3390/genes13071119)
Supplement: Supplementary file 1 [file genes-13-01119-s001.zip › Additional file S4.pdf]

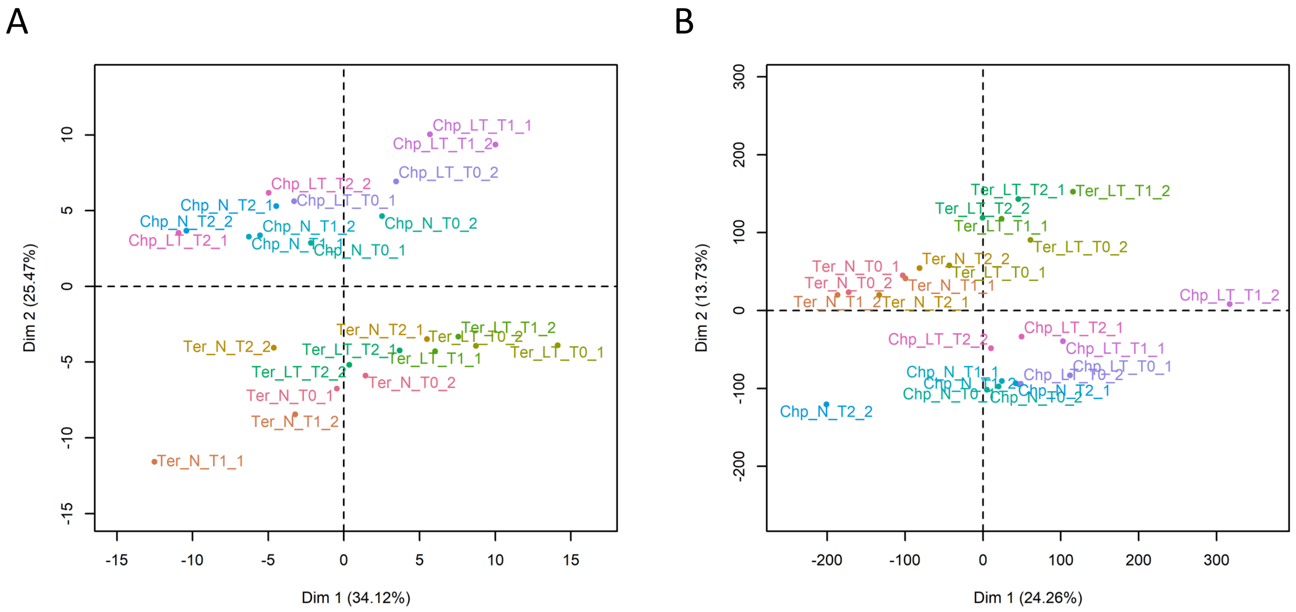

Additional file S4: Principal component analyses of miRNA (A) and mRNA (B) samples Identical colours were used to represent the biological replicates (_1 and _2 suffixes).
