# Supplementary figures and images for: Integrated sRNA-seq and RNA-seq Analyses Reveal a microRNA Regulation Network Involved in Cold Response in Pisum sativum L
Source: Genes (Basel). 2022 Jun 22;13(7):1119. doi: 10.3390/genes13071119 (PMC9322779; doi:10.3390/genes13071119)

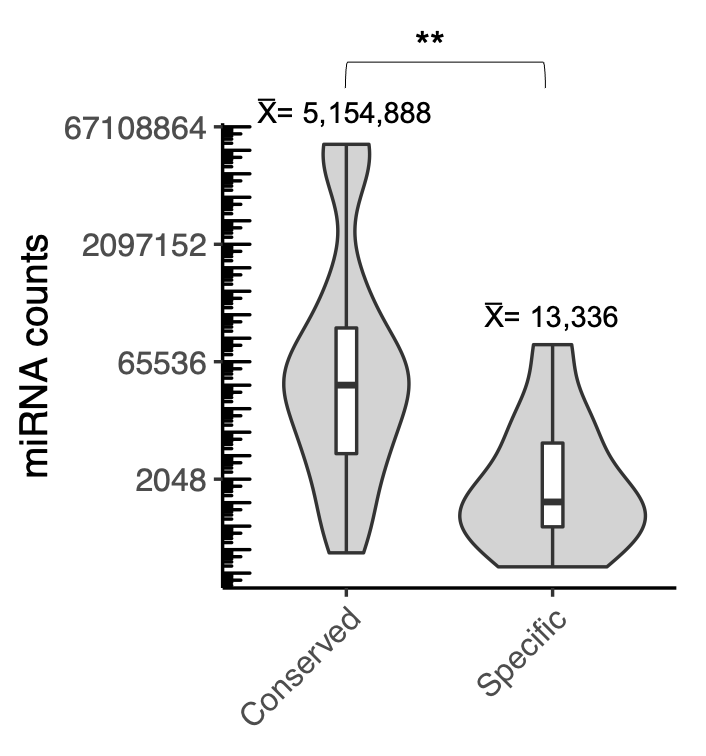

Supplement: Supplementary file 1 [file genes-13-01119-s001.zip › Additional file S3 v250322.png]
